# Cyclohexylamine, an active compound from *Toddalia asiatica*, contracts epididymal vas deferens via serotonergic receptors

**DOI:** 10.37796/2211-8039.1025

**Published:** 2020-06-05

**Authors:** Yuh-Fung Chen, Yu-Wen Wang, Ih-Sheng Chen, Huei-Yann Tsai

**Affiliations:** a Department of Pharmacology, China Medical University, Taichung, Taiwan; b School of Pharmacy, Kaohsiung Medical University, Kaohsiung, Taiwan; c Department of Pharmacy, China Medical University Hospital, Taichung, Taiwan

**Keywords:** Cyclohexylamine, primary, Serotonergic receptors, Isolated rat epididymal vas deferens, Serotonin 5-HT_2A_ subtype

## Abstract

**Background:**

*Toddalia asiatica* of Rutaceae, a Taiwan folk medicine, is used as an analgesic and anti-inflammatory herb. Cyclohexylamine (CHA) is an active compound from *T. asiatica.* Previous reports indicate CHA contracts rat vas deferens. However, the contractile mechanism of CHA on rat vas deferens was not yet reported. The purpose of this study was to investigate the contractile mechanism of CHA on rat epididymal portion of vas deferens.

**Methods:**

Male S.D. rats weighting between 200 g to 250 g were used. The epididymal portion of vas deferens was isolated and was added with various concentrations of serotonin, serotonin antagonists and CHA.

**Results:**

Serotonin elicited dose-dependent (1 × 10^−7^M~1 × 10^−4^M) contractions on rat epididymal vas deferens, which were inhibited by pretreatment with ketanserin (1 × 10^−8^ M ~ 1 × 10^−6^ M), methysergide (1 × 10^−5^ M) and propranolol (1 × 10^−4^ M), respectively. CHA elicited dose-dependent (1 × 10^−8^M~1 × 10^−4^M) contractions on rat epididymal vas deferens. The contractions of CHA (1 × 10^−4^M) on epididymal vas deferens were enhanced by serotonin in a dose-dependent manner. Methysergide (1 × 10^−7^ ~1 × 10^−5^ M) did not affect the contractions evoked by CHA. However, the CHA elicited contraction was almost completely inhibited by ketanserin (1 × 10^−5^ M) and was enhanced by propranolol. The effect of propranolol at the concentration of 1 × 10^−4^ M on CHA was likely as CHA at high concentration alone.

**Conclusions:**

From the above results, the contraction evoked by CHA on the isolated rat epididymal vas deferens might be mediated by serotonergic receptors through 5-HT_2A_ subtype.

## 1. Introduction

**Fig. 1:**
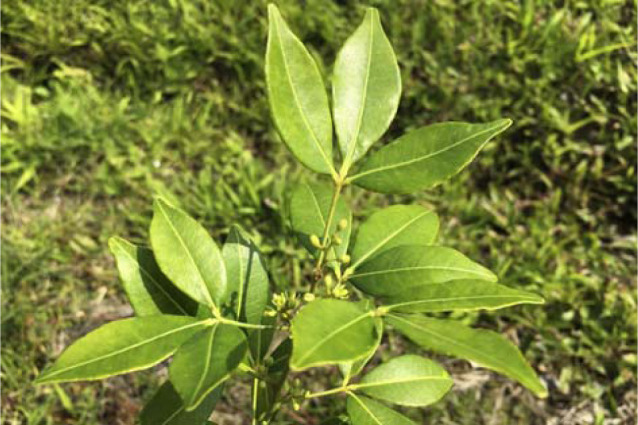
Photograph of Toddalia asiatica L. Rutaceous plant.

*Toddalia asiatica* L. (Rutaceae) (Fig. 1) is used as an analgesic and anti-inflammatory herb in Taiwan folk medicine. It processes many pharmacological activities, such as anti-platelet aggregation [1], contraction of ilea strip and aortal strip of rat [2], anti-inflammatory and anti-oxidant activities [3], induction of differentiation and apoptosis in U937 leukemic cells [4], antimalarial effect [5]. It also processes antinociceptive and anti-inflammatory effects [6], enhances differentiation and lipolysis of adipocytes [7]. Besides, it processes antimicrobial and antifungal activities [8,9,10], and anti-diabetic activity [11], analgesia for central and peripheral pain management [12]. According to our preliminary data, the methanolic extract of *T. asiatica* L. (250 mg/kg) does have anti-inflammatory and analgesic effects. The potency is equivalent to 100 mg/kg naproxen (unpublished data).

The active compounds from *T. asiatica* L. are cyclohexylamine, toddanone, brailin, dl-syringaresinol and isopimpinellin[13]. The pharmacological studies of these active ingredients reveal that cyclohexylamine (CHA) can induce contractions and enhance the norepinephrine-induced contraction on rat epididymal vas deferens. It reveals that CHA-induced contraction on rat epididymal vas deferens may be related to the regulation of the sympathetic nervous system (unpublished data). The CHA-induced contractions of rat epididymal vas deferens was blocked by phenoxybenzamine (an α_1_-adrenoceptor antagonist) and was not changed by ganglion blockade, bilateral adrenalectomy, or nephrectomy and persisted in decerebrate and spinal cats [14,15].

Serotonin (5-HT) receptors presented on rat vas deferens [16,17]. In our preliminary study, 5-HT enhanced CHA-induced contractions. The interactions between 5-HT and CHA on rat epididymal vas deferens were not yet reported. Thus, the present study aimed to investigate the relationships between CHA- and serotonergic nervous system induced-contraction on rat epididymal vas deferens.

## 2. Materials and methods

### 2.1. Materials

Cyclohexylamine was purchased from Wako, Japan. Ketanserin tartrate and methysergide male-ate were purchased from Tocris, USA. Serotonin and propranolol were bought from Sigma-Aldrich, USA. All drugs were dissolved in millipore water. The composition of Kreb's solution is expressed as follows (mM/L): NaCl 119; NaHCO_3_ 24.9; D-Glucose 11; KH_2_PO_4_ 1.2; KCl 4.6; MgSO_4_ · 7H_2_O 1.2; CaCl_2_ · 2H_2_O 1.5.

### 2.2. Ethics statement and preparations of isolated rat epididymal vas deferens

Male Sprague Dawley (SD) rats weighing between 200 and 250 g were purchased from National Laboratory Animal Center (NLAC), Taipei. Animals were fed with standard chow and housed in standard cages at a constant room temperature of 22 ± 1°C. Relative humidity 55 ± 5% with 12 h inverted light-dark cycle for at least one week prior to the experiment. The experimental protocol was approved by the Institutional Animal Care and Use Committee (IACUC), China Medical University (permit number: 2019-332). Male SD rats were deeply anesthetized by an intraperitoneal injection of 50 mg/kg of zoletil^®^ and sacrificed by cervical dislocation. The vas deferens were removed and cleaned of the surrounding connective tissue and blood vessels. Only the epididymal portions (0.6 – 1 cm) of vas deferens were used. The tissues were mounted into 5 ml Magnus-organ baths containing Kreb's solution at 37° C and bubbled with 95% O_2_/5% CO_2_. Preparations were loaded with 1.5 g resting tension, and then Kreb's solution was refreshed every 10 min. Preparations were equilibrated for 1 hr.

### 2.3. Effects of different concentrations of serotonin (5-HT) on cyclohexylamine (CHA)-induced contraction of the isolated epididymal vas deferens of rats

Different concentrations of 5-HT (1 × 10^−7^ M~1 × 10^−4^M) was administered ten minutes before CHA (1 × 10^−4^M) administration, observed and recorded the effect of 5-HT on the CHA-induced contraction of the epididymal vas deferens of rats.

### 2.4. Effects of 5-HT_2_/5-HT_1C_ serotonergic antagonist on the CHA-induced contraction of the isolated epididymal vas deferens of rats

Different concentrations of methysergide (1 × 10^−7^ M~1 × 10^−^^5^M) or ketanserin (1 × 10^−8^M~ 1 × 10^−6^M) was administered ten minutes before CHA (1 × 10^−4^M) administration, observed and recorded the effect of methysergide or ketanserin on the CHA-induced contraction of isolated epididymal vas deferens of rats.

### 2.5. Effects of 5-HT_1_ serotonergic antagonists on the CHA-induced contraction of the isolated epididymal vas deferens of rats

Different concentrations of propranolol (1 × 10^−6^M~1 × 10^−4^M) were given in advance, and the effect of propranolol on the CHA-induced contraction of the isolated epididymal vas deferens of rats was observed and recorded.

### 2.6. Effects of different concentrations of 5-HT on the isolated epididymal vas deferens of rats

Different concentrations of 5-HT (1 × 10^−7^M~1 × 10^−4^M) were administered and the contraction of vas deferens was observed and recorded. Twenty minutes later, the drug was washed out and replaced with fresh Krebs solution in every ten minutes until the contractile response returned to baseline. The next concentration experiment was then performed.

### 2.7. Effects of 5-HT_2_/5-HT_1C_ serotonergic antagonists on the 5-HT-induced contraction of the isolated epididymal vas deferens of rats

Ten minutes before 5-HT (1 × 10^−^^5^M) administration, different concentrations of ketanserin (1 × 10^−8^M~1 × 10^−6^M) or methysergide (1 × 10^−7^M~1 × 10^−^^5^M) was added to the organ bath. The effects of ketanserin or methysergide on the 5-HT-induced contraction of the isolated epididymal vas deferens of rats were recorded.

### 2.8. Effects of 5-HT_1_ serotonergic antagonists on the 5-HT-induced contraction of the isolated epididymal vas deferens of rats

Ten minutes before 5-HT (1 × 10^−^^5^M) administration, different concentrations of propranolol (1 × 10^−6^M~1 × 10^−3^M) was added to the organ bath. The effects of propranolol on the 5-HT- induced contraction of the isolated epididymal vas deferens of rats were recorded.

### 2.9. Statistical analysis

The results were expressed as mean ± S.E. The differences between mean values were compared using one-way ANOVA (post hoc test with Duncan's test) or the Student *t*-test and were considered statistically significant when P < 0.05.

## 3. Results

### 3.1. Effects of different concentrations of serotonin (5-HT) on cyclohexylamine (CHA) induced contraction of the isolated epididymal vas deferens of rats

**Fig. 2:**
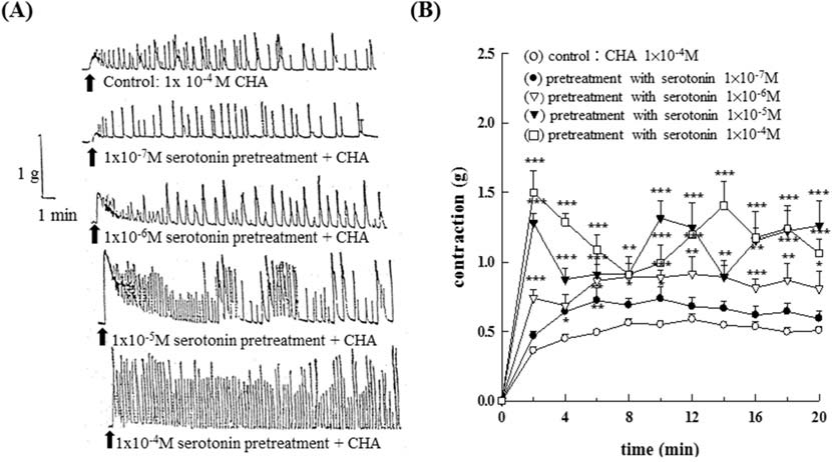
Effects of different concentrations of serotonin (5-HT) on cyclohexylamine (CHA) induced contraction of the isolated epididymal vas deferens of rats. Fig. 2A represented the contraction trace of isolated rat epididymal vas deferens. The contraction force (g) change in CHA and serotonin pretreatment in Fig. 2B. With increasing concentration of 5-HT (1 × 10^−7^M ~1 × 10^−4^M) showed a dose-dependent potentiation on CHA (1 × 10^−4^M)-induced contraction of the isolated epididymal vas deferens of rats (P < 0.05~P < 0.001).

Data were shown in Fig. 2. With increasing concentration of 5-HT (1 × 10^−7^M ~1 × 10^−4^M) showed a dose-dependent potentiation on CHA (1 × 10^−4^M)-induced contraction of the isolated epididymal vas deferens of rats (P < 0.05~P < 0.001). Fig. 2A represented the contraction trace of isolated rat epididymal vas deferens and the contraction force (g) change (Fig. 2B) in CHA and serotonin pretreatment.

### 3.2. Effects of 5-HT_2_/5-HT_1C_ serotonergic antagonist on the contraction of the isolated epididymal vas deferens of rats induced by CHA

**Fig. 3:**
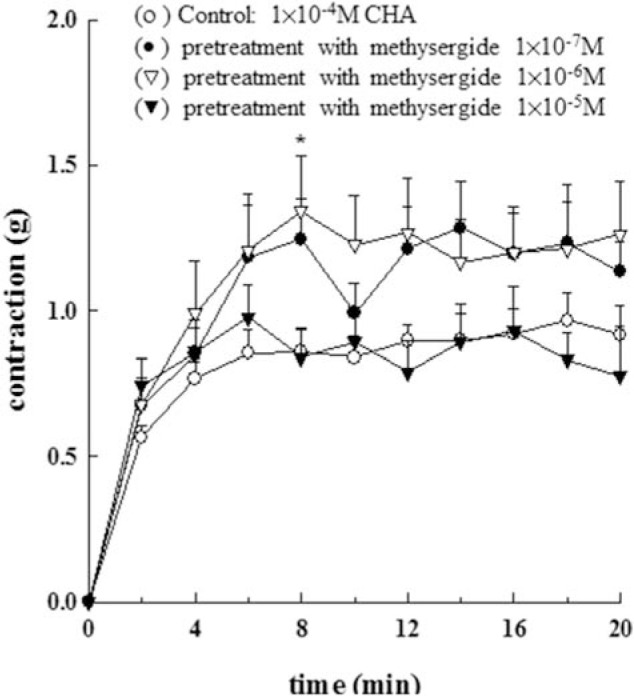
Effects of 5-HT_2_/5-HT_1C_ serotonergic antagonist, methysergide, on the contraction of the isolated epididymal vas deferens of rats induced by CHA. Different concentrations of methysergide (1 × 10^−^^7^M ~1 × 10^−^^5^ M) did not have any statistically significant effect on the CHA (1 × 10^−^^4^M)-induced isolated epididymal vas deferens of rats.

**Fig. 4:**
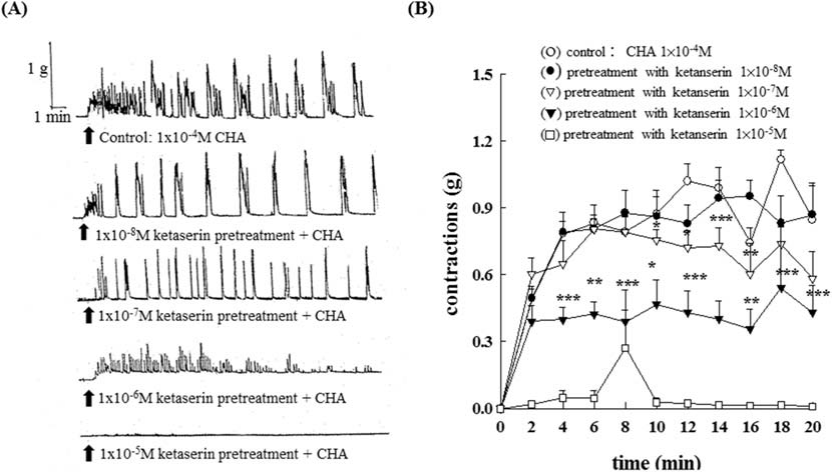
Effects of 5-HT_2_/5-HT_1C_ serotonergic antagonist, ketanserin, on the CHA-induced contraction of the isolated epididymal vas deferens of rats. Fig. 4A represented the contraction trace of isolated rat epididymal vas deferens and the contraction force (g) change in CHA and ketaserin pretreatment in Fig. 4B (P < 0.05~P < 0.001). The CHA-induced contraction of epididymal vas deferens was completely inhibited by ketanserin at the concentration of 1 × 10^−5^ M.

Different concentrations of methysergide (1 × 10^−7^M ~1 × 10^−5^ M) did not have any statistically significant effect on the CHA (1 × 10^−4^M)- induced isolated epididymal vas deferens of rats (as shown in Fig. 3). However, different concentrations of ketanserin (1 × 10^−8^M~1 × 10^−5^M) had a concentration-dependent inhibitory effect on the CHA (1 × 10^−4^M)-induced isolated epididymal vas deferens of rats (as shown in Fig. 4). Fig. 4A represented the contraction trace of isolated rat epididymal vas deferens and the contraction force (g) change in CHA and ketaserin pretreatment in Fig. 4B (P < 0.05~P < 0.001). The CHA-induced contraction of epididymal vas deferens was completely inhibited by ketanserin at the concentration of 1 × 10^−5^ M.

### 3.3. Effects of 5-HT_1_ serotonergic antagonists on the contraction of the isolated epididymal vas deferens of rats induced by CHA

**Fig. 5:**
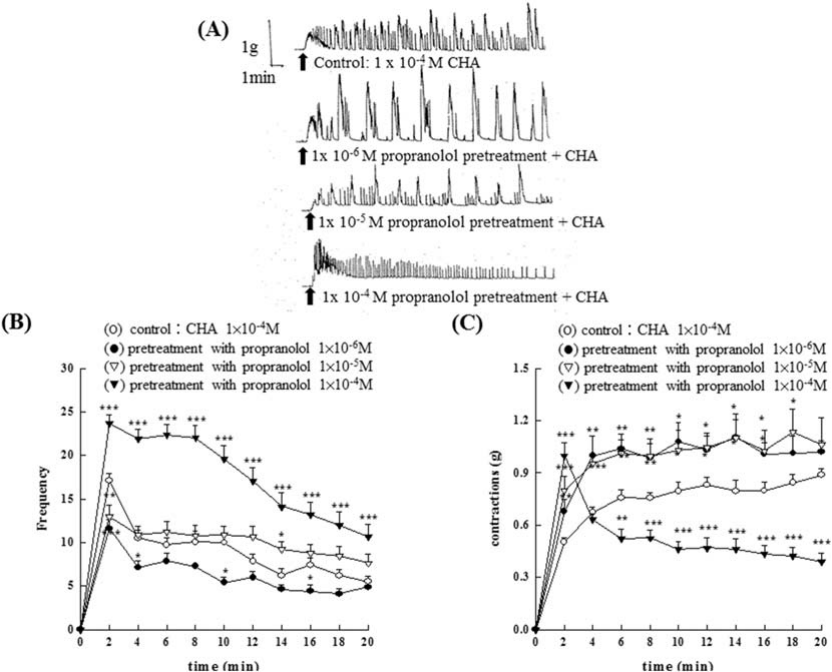
Effects of 5-HT_1_ serotonergic antagonists on the contraction of the isolated epididymal vas deferens of rats induced by CHA. With the increase of the time, propranolol (1 × 10^−5^M and 1 × 10^−6^M) enhances the CHA-induced contraction of the vas deferens. Fig. 5A represented the contraction trace of isolated rat epididymal vas deferens, the frequency change (Fig. 5B), and the contraction force (g) change in CHA and propranolol pretreatment in Fig. 5C. Propranolol at the concentration of 1 × 10^−4^M showed a suppression effect on CHA-induced contraction (P < 0.01) and the frequency of contraction increased significantly (P < 0.01).

Results were shown in Fig. 5. Pretreatment with different concentrations of propranolol (1 × 10^−6^M~1 × 10^−4^M) showed a concentration- dependent enhancement of CHA (1 × 10^−4^M)- induced contraction of the rat epididymal vas deferens at the first two minutes. With the increase of the time, propranolol (1 × 10^−5^M and 1 × 10^−6^M) enhanced the CHA-induced contraction of the vas deferens. However, propranolol at the concentration of 1 × 10^−4^M showed a suppression effect on CHA-induced contraction (P < 0.01) and the frequency of contraction increased significantly (P < 0.01), the results were shown in Fig. 5. Fig. 5A represented the contraction trace of isolated rat epididymal vas deferens, the frequency change (Fig. 5B), and the contraction force (g) change in CHA and propranolol pretreatment in Fig. 5C.

### 3.4. Effects of different concentrations of 5-HT on the isolated epididymal vas deferens of rats

**Fig. 6:**
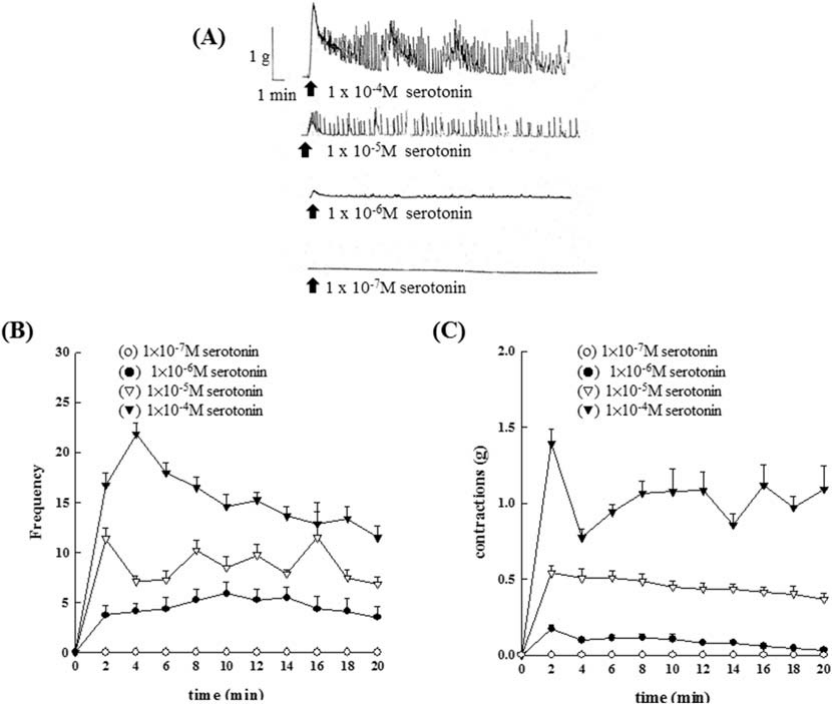
Effects of different concentrations of 5-HT on the isolated epididymal vas deferens of rats. 5-HT (1 × 10^−7^M~1 × 10^−4^M) showed a dose-dependent increase in the contraction amplitude (6A), frequency (6B) and the contraction force (6C) of the rat epididymal vas deferens. The maximum contraction tension was 0.17 ± 0.03, 0.54 ± 0.05, and 1.39 ± 0.09, respectively.

As shown in Fig. 6, 5-HT (1 × 10^−7^M~1 × 10^−4^M) showed a dose-dependent increase in the contraction amplitude (Fig. 6A), frequency (Fig. 6B) and the contraction force (Fig. 6C) of the rat epididymal vas deferens. The maximum contraction tension was 0.17 ± 0.03, 0.54 ± 0.05, and 1.39 ± 0.09, respectively. However, the concentration of 5-HT at 1 × 10^−7^ M did not contract rat epididymal vas deferens.

### 3.5. Effects of 5-HT_2_/5-HT_1C_ serotonergic antagonists on the contraction of the isolated epididymal vas deferens of rats induced by 5-HT

**Fig. 7:**
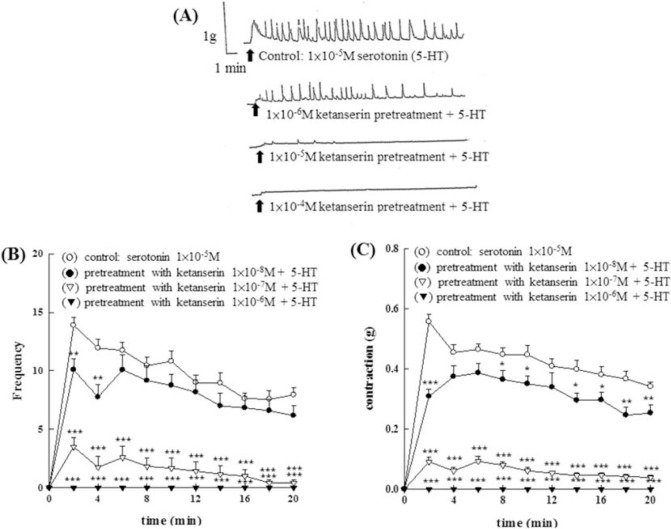
Effects of 5-HT_2_/5-HT_1C_ serotonergic antagonist, ketanserin, on the contraction of the isolated epididymal vas deferens of rats induced by 5-HT. The effect of pretreatment of different concentrations of ketanserin (1 × 10^−^^8^M~1 × 10^−6^M) showed a dose-dependent inhibition of 5-HT (1 × 10^−5^M) induced contraction in amplitude (7A, 7C) and frequency (7B).

**Fig. 8:**
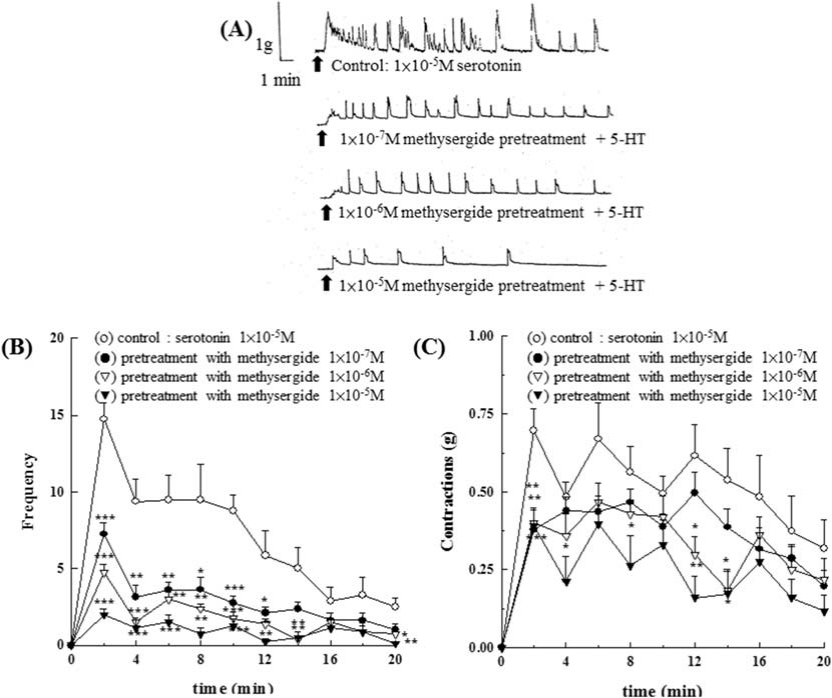
Effects of 5-HT_2_/5-HT_1C_ serotonergic antagonist, methysergide, on the contraction of the isolated epididymal vas deferens of rats induced by 5- HT. Pretreatment with different concentrations of methysergide (1 × 10^−7^M~1 × 10^−5^M) showed a dose-dependent inhibition of 5-HT (1 × 10^−5^M) induced contraction in amplitude (8A, 8C) and frequency (8B).

As shown in Fig. 7, the effect of pretreatment of different concentrations of ketanserin (1 × 10^−8^M~1 *×* 10^−6^M) showed a dose-dependent inhibition of 5-HT (1 × 10^−5^M) induced contraction in amplitude (Fig. 7A and C) and frequency (Fig. 7B). As shown in Fig. 8, the effect of pretreatment of different concentrations of methysergide (1 × 10^−7^M~1 × 10^−5^M) showed a dose-dependent inhibition of 5-HT (1 × 10^−5^M) induced contraction in amplitude, force (Fig. 8A and C) and frequency (Fig. 8B).

### 3.6. Effects of 5-HT_1_ serotonergic antagonist on the contraction of the isolated epididymal vas deferens of rats induced by 5-HT

**Fig. 9:**
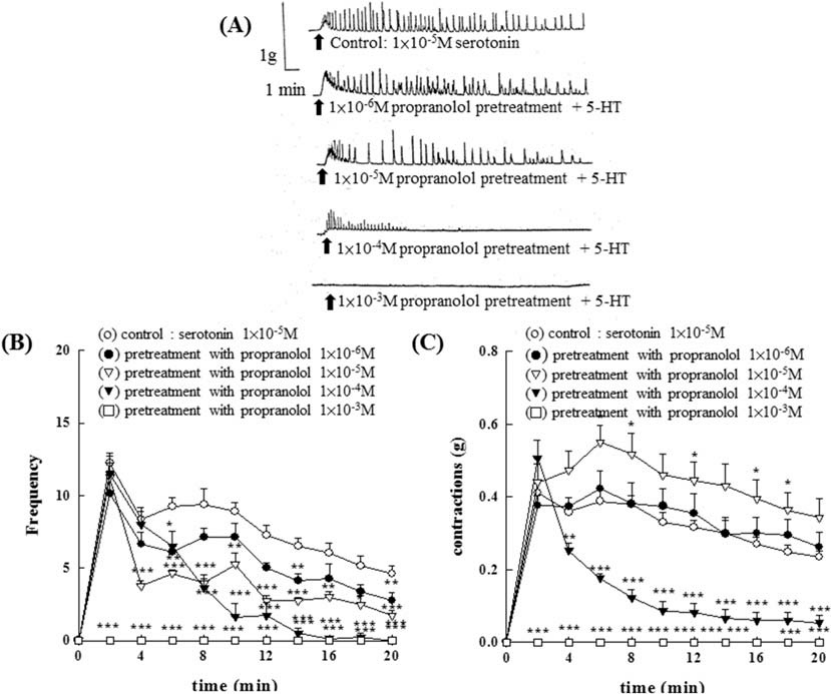
Effects of 5-HT_1_ serotonergic antagonist, propranolol, on the contraction of the isolated epididymal vas deferens of rats induced by 5-HT. At a concentration of 1 × 10^−5^M, propranolol showed a slight increase in contraction of the rat epididymal vas deferens produced by 5-HT (1 × 10^−5^M) (as shown in Fig. 9). However, when the propranolol concentration is higher than 1 × 10^−4^M, the vas deferens contraction effect produced by 5-HT (1 × 10^−5^M) will be weakened.

At a concentration of 1 × 10^−5^M, propranolol showed a slight increase in contraction of the rat epididymal vas deferens produced by 5-HT (1 × 10^−5^M) (as shown in Fig. 9). However, when the propranolol concentration is higher than 1 × 10^−4^M, the vas deferens contraction effect produced by 5- HT (1 × 10^−5^M) will be weakened, as shown in Fig. 9. When given propranolol (1 × 10^−3^ M), the contractile response of 5-HT was completely suppressed. In addition, the statistical analysis showed that different concentrations of propranolol (1 × 10^−6^M to 1 × 10^−3^M) can inhibit the frequency of vas deferens produced by 5-HT, as shown in Fig. 9.

## 4. Discussion

Cyclohexylamine (CHA) is one of the active ingredients of *T. asiatica* L. [13]. However, CHA is also the main metabolite of cyclamate. Cyclamate, an FDA approved artificial sweetener [18], will be metabolized to CHA by entero-bacteria [19–21]. Ingestion of CHA or high dose of a mixture of cyclamate and saccharin will induce bladder tumors in rats [22–25]. CHA acts on Sertoli cells of the testis and causes testicular atrophy in the rat [22]. The effects of CHA on the testis attributed to the direct action of CHA on seminiferous epithelium [26].

There are few reports on the effects of CHA on vas deferens, and the action mechanism of CHA is not yet precise. According to our data, the contraction caused by CHA involved in the control of the sympathetic nervous system. CHA can directly act on post-synaptic adrenergic α_1A_ and α_1B_ receptors. Besides, CHA also directly acts on presynaptic adrenal receptors, thus releasing endogenous catecholamine. The effects of CHA on the contraction of the epididymal vas deferens are all calcium-dependent responses (unpublished data). Besides the role of the sympathetic nerve, whether there are other action mechanisms also involved in this contraction of CHA is the primary goal of this study.

Many kinds of literature indicate that there are serotonergic receptors on the vas deferens of rats [27–30]. Therefore, we use serotonin to observe the contractile response of the epididymal vas deferens produced by CHA. Serotonin enhanced the contractile response of CHA. This result suggests that serotonergic receptors may be involved in CHA- induced contraction response of epididymal vas deferens.

Comparing the response of noradrenaline (NA) and 5-HT on rat vas deferens, the contractile response caused by NA is fast, and tonic; and 5-HT first produces rapid contraction, accompanied by a phasic and rhythmic contraction response (phasic and rhythmic), followed by a tonic contraction. Moreover, the vasoconstriction response of rats induced by 5-HT is 15 times weaker than that of NA [31]. Serotonin-induced contraction of vas deferens in rats can be divided into direct and indirect responses; direct contractile responses are related to the direct activation of tryptaminergic and α- adrenergic receptors [32,33]. Serotonin is a medium that is accepted by the presynaptic tryptaminergic receptor, and thus promotes the release of NA from the end of the sympathetic nerve, which causes vasocontraction. Also, the direct contractile response of serotonin is also related to the mediator of the α_1_-adrenoceptor. As for the indirect contraction response of serotonin, the release of NE is involved, and this effect is the most crucial reason for the contraction of the vas deferens [34].

The rapid contraction produced by 5-HT is mainly mediated by the post-synaptic 5-HT_2_ neurokinin receptors (5-HT_2_ receptors). While the part of the tonic contraction is not only mediated by post-synaptic serotonin receptors (5-HT_2_ receptors) but also related to the action of NE released from neuronal stores [35]. However, high doses of ketanserin can completely inhibit the contraction caused by serotonin, mainly because the 5-HT_2_ neurokinin receptor mediates the contraction of rat vas deferens caused by serotonin [32,35]. In addition to the 5-HT_2_ neurokinn blocking effect, ketanserin also has the activity of antagonizing α-adrenal receptors at a high concentration [36]. Similar results founded in our experiments that different concentrations of ketanserin attenuated the dose-dependent reduction of serotonin-induced contraction of the vas deferens of the epididymal vas deferens of the rat, and ketanserin at high doses also showed complete inhibition of serotonin. Besides, methysergide inhibits the effect of serotonin is smaller than that of ketanserin. Methysergide has an antagonistic effect only at high concentrations, which may be related to the partial agonist of methysergide itself. Methysergide belongs to the 5HT_2_/5-HT_1c_ neurokinin antagonist [37], it is possible that the blocking effect of 5-HT_1c_ neurokinin by methysergide.

Studying the effect of serotonin on the twitch- response contraction of the mice vas deferens produced by electrical stimulation [38], it was found that serotonin produces a “bell-shape” concentration response curve below 10^−8^ M to 10^−4^ M. When the concentration is 10^−5^ M, serotonin can achieve the maximum contraction-enhancing effect. While the concentration of serotonin is higher than 10 ^−5^ M, it shows a contraction-inhibitory effect. Therefore, serotonin initially enhance the vas deferens response of mice induced by electrical stimulation [38].is because of the deactivation of the 5-HT_2_ neurokinin receptors on post-synaptic sites [32,38,39], resulting in an increased contractile response. The inhibitory response at higher concentrations of serotonin relates to the activation of presynaptic 5-HT_1_ neurokinin receptors (especially 5-HT_1A_ neurokinamine receptors), but it is not excluded that Results of involvement of postnatal 5- HT neurokinin receptors [32].

Propranolol, a non-selective 5-HT_1_ neurokinin antagonist, at low concentrations enhances the serotonin-induced contraction of epididymal vas deferens of the rat. The inhibition of presynaptic 5- HT_1_ neurokinin receptors may enhance the contractile effect of post-synaptic 5-HT_2_ neurokinin receptors. The reason why high doses of propranolol completely inhibit the contractile response produced by serotonin is not known, and further investigation is needed. Perhaps, there may be other mechanisms (such as β-sympathetic receptors) that are also involved in serotonin's effect on isolated rat contractile response of the epididymal vas deferens.

However, neither the selective 5-HT_2_ antagonist - LY 53857 nor the non-selective 5-HT antagonist -1- NP could block the vas deferens caused by serotonin [34]. Thus, the contractile effect of serotonin is mainly mediated by α-adrenergic receptors, not by serotonergic receptors. However, the role of serotonin is not excluded for NE is released from neurons and results in a contractile response [34].

Although the actual contraction mechanism of serotonin induced vas deferens is still controversial, its mechanism is mainly related to the mediator of the post-synaptic neurokinin receptor combined with NE that released from nerve terminal. In our experiments, different concentrations of 5-HT_2_/5-HT_1c_ neurokinin antagonist-ketanserin produced the dose-dependent inhibition of CHA-induced contractile responses of the rat epididymal vas deferens. It revealed the effect of cyclohexylamine is related to the regulation of neurokinin receptors. Ketanserin is a selective 5-HT_2A_ neurokinin antagonist [40], and neurokinin involves in CHA-induced contraction, especially the 5-HT_2A_ neurokinin receptor. However, the involvement of neurokinin receptors in the CHA-induced contraction may also be an indirect effect. Because different concentrations of methysergide, 5-HT_2_/5-HT_1c_ neurokinin antagonist, do not affect the response of CHA. Propranolol can enhance the contraction of CHA at concentrations of 1 × 10^−6^ M and 1 × 10^−5^ M, but at the concentration of 1 × 10^−4^ M appears to suppress the contraction amplitude of CHA. However, the effect of increased contraction frequency can only be deduced that perhaps the effect of cyclohexylamine on the contraction of the epididymal vas deferens may also be related to the 5-HT_1c_ neurokinin receptors and β-sympathetic receptors.

## 5. Conclusions

**Fig. 10:**
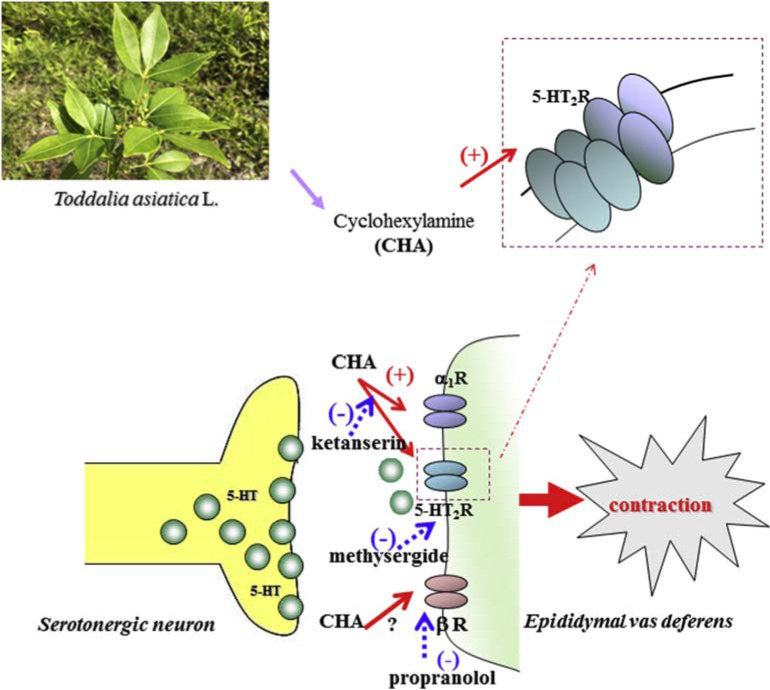
Proposed action mechanism of cyclohexylamine (CHA)-induced contraction of epididymal vas deferens of rat. CHA induced contraction on the isolated rat epididymal vas deferens may be partially mediated by serotonergic neuron.

From the above results, CHA induced contraction on the isolated rat epididymal vas deferens might be mediated by serotonergic receptors especially through 5-HT_2A_ subtype. The proposed action mechanism of cyclohexylamine (CHA)-induced contraction on epididymal vas deferens of rat shows in Fig. 10).
